# The pivotal role of osteopontin in UV-induced skin inflammation in a mouse model

**DOI:** 10.1098/rsob.230397

**Published:** 2024-11-13

**Authors:** Haesoo Kim, Chang-Yup Shin, Chi-Hyun Park, Dong Hun Lee, Si-Hyung Lee, Jin Ho Chung

**Affiliations:** ^1^ Department of Dermatology, Seoul National University College of Medicine, Seoul, Republic of Korea; ^2^ Laboratory of Cutaneous Aging Research, Biomedical Research Institute, Seoul National University Hospital, Seoul, Republic of Korea; ^3^ Institute of Human-Environmental Interface Biology, Medical Research Center, Seoul National University, Seoul, Republic of Korea; ^4^ Institute on Aging, Seoul National University, Seoul, Republic of Korea

**Keywords:** osteopontin, inflammation, photoaging, ultraviolet light

## Abstract

Osteopontin (OPN) is a pro-inflammatory protein that influences bone remodelling, wound healing, angiogenesis, allergic inflammation, and skin diseases such as psoriasis, contact dermatitis and skin cancer. However, the role of OPN in the skin remains unclear. Therefore, this study aimed to investigate the role of OPN in the skin, particularly in the context of ultraviolet (UV) irradiation-induced inflammation. OPN expression and its effects on inflammatory modulators were assessed in human skin, in a mouse model and *in vitro*, using a UV source emitting both UVB and UVA radiation, which collectively contribute to UV-induced skin inflammation. OPN expression increased in human and mouse skin after UV irradiation. Compared with wild-type mice, UV irradiation-induced skin phenotypes, such as erythema and skin thickening, were alleviated in OPN^−/−^ mice. In addition, the number of immune cells recruited to the skin after UV irradiation and the expression of inflammatory cytokines and matrix metalloproteinases (MMPs) were observed to be decreased in the skin of OPN^−/−^ mice compared with that of wild-type mice. By contrast, the degree of skin inflammation was higher in the hOPN KI mice than in wild-type mice. Treatment with recombinant OPN increased the expression of MMP-1 and inflammatory cytokines in human dermal fibroblasts and epidermal keratinocytes *in vitro*. Our results suggest that OPN may play a regulatory role in UV-induced skin inflammation.

## 1. Introduction

Skin serves as a barrier between the internal and external environments of an organism and protects it from harmful environmental substances or pathogens. Both intrinsic and extrinsic factors induce physiological changes in the skin. Inflammation is a complex biological response that can cause swelling, redness, itching, pain and loss of skin function [1]; dysregulation of inflammation can lead to various skin diseases. Ultraviolet (UV) light, particularly UVB (280−320 nm) and UVA (320−400 nm) radiation, is the main cause of inflammatory skin damage. Exposure to UVB radiation primarily causes DNA damage and direct skin inflammation, while UVA radiation penetrates deeper into the skin, leading to oxidative free radical damage to DNA and other macromolecules, as well as inflammation. UVC (200−290 nm), having the shortest wavelength, is the most harmful to the skin but is blocked by the ozone layer. Both UVB and UVA irradiation can lead to many pathophysiological disorders, including autoimmune reactions, photoaging and skin cancer [1,2].

Osteopontin (OPN), also known as secreted phosphoprotein-1, is a small integrin-binding N-linked glycoprotein. OPN can act as a secreted cytokine (sOPN) or as an intracellular protein (iOPN) [3–5]. Its molecular weight ranges from 30 to 100 kDa, depending on post-translational modifications [6]; the initial peptide is cleaved at multiple sites to produce CD44- and integrin-binding domains. By interacting with CD44 and integrins, sOPN mediates cell migration and adhesion [7], exerts anti-apoptotic effects and possesses T helper 1 (Th1) cytokine functions [8]. iOPN regulates macrophage migration and interferon-alpha secretion from plasmacytoid dendritic cells [9].

Under normal conditions, OPN is expressed at low levels in many cell types, including osteoblasts, smooth muscle cells, macrophages, endothelial cells and fibroblasts [3]. However, a previous study revealed that OPN expression increased significantly in pathological conditions, such as neurodegenerative diseases, bacterial infections, autoimmune diseases and carcinogenesis [10]. In addition, OPN is reportedly associated with inflammation, diseases and cancers of the skin. OPN levels are elevated in autoimmune diseases such as systemic lupus erythematosus (SLE) [11] and psoriasis [12]. Serum levels of OPN were elevated in patients with SLE, and OPN enhanced Th1-mediated inflammatory processes involved in the exacerbation of SLE. Moreover, OPN is involved in the pathophysiology of psoriasis through the upregulation of Th1- and Th17-mediated inflammation [13]. OPN is also associated with skin cancer progression; it inhibits apoptosis in mouse melanoma cells and induces adhesion, migration and survival of tumour cells [14]. OPN overexpression promotes tumorigenesis and metastasis of squamous cell carcinoma and melanoma by inhibiting tumour cell apoptosis; OPN expression is a possible prognostic marker for melanoma [15–18].

Important information regarding OPN function has been obtained through the analysis of OPN-knockout (OPN^−/−^) mice. In injury models, OPN^−/−^ mice show changes in collagen structure, including a smaller diameter of collagen fibrils, in healed wounds due to an abnormality in collagen fibrillogenesis [19]. In addition, owing to a decrease in macrophage activity, an increase in the spillage of necrotic cell debris or intracellular contents at the injury site has been observed. By contrast, OPN is involved in various aspects of the immune response and contributes to the resistance to infection [20]. In some cases, such as renal ischaemia and rotavirus infection, OPN deficiency leads to greater tissue damage; however, in tumorigenesis or inflammation, the presence of OPN has more harmful consequences [17]. Thus, OPN has both protective and detrimental effects on the body [17].

This study aimed to investigate the role of OPN as an inflammatory cytokine in the skin, particularly in the context of UV-induced skin inflammation.

## 2. Material and methods

### 2.1. Human skin samples

Skin samples were obtained from healthy volunteers. An F75/85W/UV21 fluorescent lamp (emission range: 285−350 nm; peak: 310−315 nm, Philips, Amsterdam, Netherlands) was used to irradiate UVA and UVB. The distribution of its power output was 56.7% UVB (280−320 nm), 42.8% UVA (320−400 nm) and 0.5% UVC (<280 nm). UVC light (below 290 nm) was blocked using a filter. The amount of UV radiation that produces minimal erythema was defined as the MED, depending on an individual’s skin sensitivity. The MED was determined 24 h after UV irradiation and typically ranges from 40 mJ cm^−2^ to 70 mJ cm^−2^. The buttock skin was irradiated with 2 MED of UV light and sun-protected and UV-irradiated skin samples were obtained at the indicated times by punch biopsy.

### 2.2. Cell culture and recombinant OPN treatment

Primary NHDFs and NHEKs were isolated from human foreskin tissues. NHDFs were cultured in Dulbecco’s modified Eagle’s medium (DMEM, Welgene, Daegu, Korea) supplemented with 10% foetal bovine serum (Gibco, Rockville, MD, USA) and 1% penicillin/streptomycin (Gibco), while NHEKs were cultured in EpiLife medium supplemented with human keratinocyte growth supplement (Thermo Fisher Scientific, Inc. Rockford, IL, USA). Cells were grown in a humidified atmosphere with 5% CO_2_ at 37°C. For experiments, cells were seeded at a density of 2 × 10^5^ in 35 mm dishes and grown overnight. After starvation in serum-free media for 24 h, the cells were treated with rOPN (0, 20, 100 and 200 ng ml^−1^). The rOPN was purchased from Bio-Legend (San Diego, CA). The conditioned medium and cell lysates were collected 72 h after treatment.

### 2.3. Western blotting

Cells were lysed in cell lysis buffer (BD Biosciences, Franklin Lakes, NJ, USA) mixed with a protease inhibitor mixture (Roche Applied Science, Penzberg, Germany) and phosphatase inhibitor mixture (Sigma-Aldrich, St. Louis, MO, USA). Equal amounts of protein were separated using 8% sodium dodecyl-sulfate polyacrylamide gel electrophoresis and transferred onto a nitrocellulose membrane (GE Healthcare Life Sciences, Buckinghamshire, UK). The membranes were probed with antibodies against MMP-1 (Lab Frontier, Seoul, Korea) and glyceraldehyde 3-phosphate dehydrogenase (GAPDH) (Cusabio, Houston, TX, USA). Immunoreactive proteins were visualized using an enhanced chemiluminescence detection system (Biomax Co., Ltd., Seoul, Korea). The band intensity was measured using ImageJ software (NIH, Bethesda, MD, USA).

### 2.4. Mice

C57BL/6-Spp1^tm1Blh(−/−)^/J (OPN^−/−^) mice purchased from Jackson Laboratory (Bar Harbor, ME, USA) were a generous gift from the laboratory of Professor Dae-Yong Kim (Seoul National University College of Veterinary Medicine). A targeting vector was designed to replace exons 4–7 of the secreted phosphoprotein 1 (*Spp1*) gene with a neomycin resistance cassette, thereby generating OPN^−/−^ mice. Herein, 8-week-old WT and OPN^−/−^ mice were used for experiments. hOPN KI mice were a generous gift from the laboratory of Professor Byung-Chul Oh (Gachon University College of Medicine). The hOPN KI mice were generated by inserting the hOPN-V5-eGFP construct into the endogenous ROSA26 locus via homologous recombination. Therefore, the mice expressed human OPN throughout their body. In this study, 8-week-old WT and hOPN KI mice were used. OPN^−/−^ mice and hOPN KI mice were backcrossed on a C57BL/6 background. All the mice were housed in cages under specific pathogen-free conditions.

### 2.5. UV irradiation on mice

The dorsal skin was shaved and chemically depilated using NICREAN cream (ILDONG Pharmaceutical Co., Seoul, Korea) 48 h before acute UV irradiation. The mice were assigned to three groups (*n* = 5 per group): non-irradiated control and UV-irradiated groups (biopsied after 48 or 72 h). UV light was applied to the dorsal skin under anesthesia (4% isoflurane). The mice were exposed to UV irradiation at a distance of 21 cm between the dorsal skin and the light source (Philips TL20W/12RS sun lamps with a filter blocking UVC below 290 nm, emission range 285−350 nm) for 4 min and 16−38 s (200 mJ cm^−2^), depending on the intensity. The intensity of UV light was measured using a UV meter (model No. 585100, Waldmann Co.). At the indicated time (48 or 72 h) after UV exposure, the mice were euthanized, and the dorsal skin was isolated and paraffin-embedded for immunostaining or lysed in protein lysis buffer or RNA extraction reagent.

### 2.6. Tissue preparation and immunohistology

Paraffin-embedded skin tissue sections (4 μm thickness) were deparaffinized and rehydrated using an ethanol gradient. Sections were subjected to heat-induced antigen retrieval in 0.01 M citrate buffer (pH 6.0). After blocking with a blocking solution for 30 min at room temperature, the sections were incubated with the primary antibody against OPN (ab22952-1-AP, Proteintech, Rosemont, IL, USA; ab8448, Abcam, Cambridge, UK), Gr-1 (108407, Biolegend) and F4/80 (ab6640, Abcam) overnight at 4℃, and further incubated with secondary antibodies fluorescently labelled with AlexaFluor 594 and AlexaFluor 488 (Invitrogen, Waltham, MA, USA) after washing. Nuclei were counterstained with 4′,6-diamidino-2-phenylindole (DAPI) and the sections were mounted in Faramount aqueous mounting medium (Dako, Carpinteria, CA, USA). Immunofluorescence images were captured using a confocal microscope (Leica STED CW; Leica Microsystems). To determine the percentage of Gr-1-positive and F4/80-positive cells, the total cells were counted following nuclear counterstaining with DAPI in more than six random fields per group.

### 2.7. RNA isolation and qRT-PCR

Skin tissues were lysed using the RNAiso Plus reagent (Takara Bio Inc., Shiga, Japan), and total RNA was isolated according to the manufacturer’s protocol. cDNA was synthesized from 1 μg of total RNA using the RevertAid First Strand cDNA Synthesis Kit (Thermo Fisher Scientific) and quantified using SYBR Premix Ex Taq (Takara Bio Inc.) with carboxy-X-rhodamine using a 7500 Real-Time PCR system (Applied Biosystems, Foster City, CA, USA) and the respective primer pairs (table 1). Expression values were normalized to the expression level of *GAPDH* mRNA.

**Table 1.** Primer sequences used for qRT-PCR.

gene	primers (5'−3')	
***hOPN***	F	TAGGCATCACCTGTGCCATACC
	R	TTGGAAGGGTCTGTGGGGCTA
***h36B4***	F	TCGACAATGGCAGCATCTAC
	R	TGATGCAACAGTTGGGTAGC
***IL-6***	F	GCAGATGAGTACAAAAGTCC
	R	GCAGAATGAGATGAGTTGTC
***CXCL1***	F	AGTGGCACTGCTGCTCCT
	R	AGCTTTCCGCCCATTCTT
***MCP-1***	F	GCTCATAGCAGCCACCTTCATTC
	R	GGACACTTGCTGCTGGTGATTC
***Gapdh***	F	GATGCCCCCATGTTTGTG
	R	ACA ACCTGGTCCTCAGTG
***mOpn***	F	GTCTGGAGAACATGGGTGCT
	R	GGGTGCAGGCTGTAAAGCTA
***Il-6***	F	GCTACCAAACTGGATATAATCAGGA
	R	CCAGGTAGCTATGGTACTCCAGAA
***Il-10***	F	CCAAGCCTTATCGGAAATGA
	R	TTTTCACAGGGGAGAAATCG
***Cxcl1***	F	GCT GGG ATT CAC CTC AAG AA
	R	TCT CCG TTA CTT GGG GAC AC
***Cxcl2***	F	AGTGAACTGCGCTGTCAATG
	R	TCCAGGTCAGTTAGCCTTGC
***Cxcl10***	F	ATCATCCCTGCGAGCCTATCCT
	R	GACCTTTTTTGGCTAAACGCTTTC
***Mmp-9***	F	TTGAGTCCGGCAGACAATCC
	R	CCTTATCCACGCGAATGACG
***Mmp-13***	F	CATCCATCCCGTGACCTTAT
	R	GCATGACTCTCACAATGCGA
***Mcp-1***	F	CATCCACGTGTTGGCTCA
	R	GATCATCTTGCTGGTGAATGAGT

### 2.8. ELISA

Capture antibodies (mOPN, 0.4 μg ml^−1^, AF808, R&D Systems, Minneapolis, MN, USA; hOPN, 0.4 μg ml^−1^, MAB14332, R&D Systems; IL-6, 1 μg ml^−1^, 504502, Biolegend; CXCL1, 1 μg ml^−1^, MAB453, R&D Systems) were diluted in a 50 mM carbonate-bicarbonate buffer (pH 9.6). Then, 50 μl of coating buffer was added into each well of 96-well Maxisorp microplates (#468667, Thermo Fisher Scientific) and incubated overnight at 4°C. The wells were washed with washing buffer (0.05% Tween 20 in phosphate buffered saline (PBS)) and blocked with blocking buffer (1% bovine serum albumin in PBS) for 1 h at room temperature. Standards (mOPN, 2 ng ml^−1^, 763602, BioLegend; hOPN, 20 ng ml^−1^, 557102, BioLegend; mouse IL-6, 20 ng ml^−1^, 575702, BioLegend; mouse CXCL1, 10 ng ml^−1^, 453-KC, R&D Systems) and samples were mixed with the blocking buffer and added to the wells and incubated for 2 h at room temperature. Plates were washed three times with washing buffer and incubated for 1 h with 50 μl of biotinylated antibody solutions (mOPN, 0.4 μg ml^−1^, BAF808, R&D Systems; hOPN, 0.2 μg ml^−1^ BAF1433, R&D Systems; IL-6, 1 μg ml^−1^, 504602, Biolegend; CXCL1, 0.1 μg ml^−1^, BAF453, R&D Systems). Streptavidin-horseradish peroxidase (N100, Thermo Fisher Scientific) was added after washing and incubated for 30 min at room temperature. After washing with the washing buffer, 50 μl of tetramethylbenzidine substrate solution (SB01, Thermo Fisher Scientific) was added into each well. Then, 50 μl of 1.8 N H_2_SO_4_ were added to stop reactions and the absorbance was measured at 450 nm using a microplate reader (VersaMax, Molecular Devices Corporation, San Jose, CA, USA).

### 2.9. Statistical analysis

Statistical analyses were performed using the GraphPad Prism software v. 5.0.3 (GraphPad Prism, La Jolla, CA, USA). Data are presented as mean ± standard errors of mean. Statistical significance was determined using the Wilcoxon signed-rank test, two-tailed Student’s *t*‐test or Mann–Whitney *U*-test. Statistical significance was set at *p* < 0.05.

## 3. Results

### 3.1. UV irradiation increased OPN expression in the human skin

**Figure 1.**
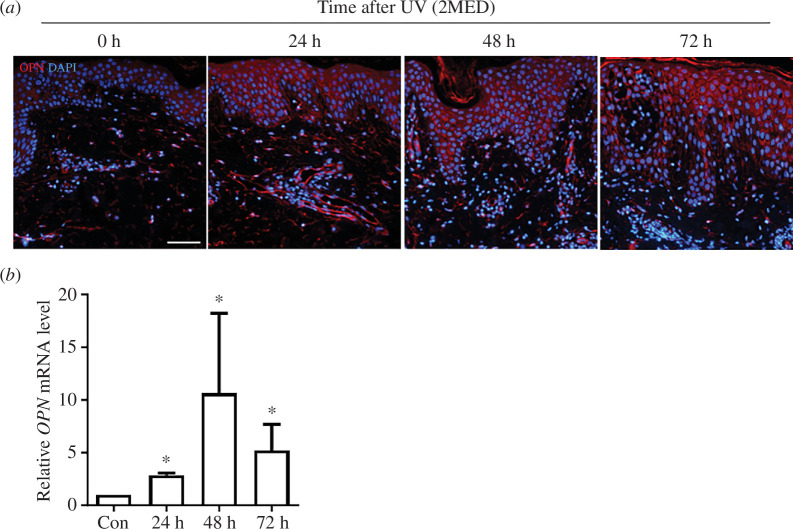
UV irradiation increases OPN expression in the human skin. Sun-protected buttock skin was irradiated with UV (2 MED) and biopsied at the indicated timepoints following UV irradiation (*n* = 4). (*a*) Representative images of OPN immunofluorescence staining (red). The nuclei stained with DAPI. Scale bars, 100 μm. (*b*) *OPN* mRNA expression in human skin tissue (*n* = 4) at the indicated timepoints after UV irradiation analysed using qRT-PCR. The lower layer of the dermis and adipose tissue of the skin were removed and analysed. Data are presented as mean ± standard errors of mean, *p < 0.05. *p*-values were calculated using the Wilcoxon signed-rank test. UV, ultraviolet; OPN, osteopontin; MED, minimal erythema dose; DAPI, 4′,6-diamidino-2-phenylindole; qRT-PCR, quantitative real-time polymerase chain reaction.

First, the effect of UV irradiation on OPN expression in human skin was evaluated. Sun-protected buttock skins were irradiated with two minimal erythema dose (MED) of UV light and biopsied 24, 48 and 72 h after irradiation. Immunofluorescence staining showed that OPN protein expression was markedly elevated in epidermal keratinocytes in the spinous layer and dermal cells in the papillary and reticular dermis after UV irradiation (figure 1*a*). In addition, *OPN* mRNA levels increased significantly 24, 48 and 72 h after UV irradiation (figure 1*b*). These data show that acute UV irradiation increases OPN expression in human skin.

### 3.2. OPN knockout mice exhibit reduced UV-induced skin inflammation

**Figure 2.**
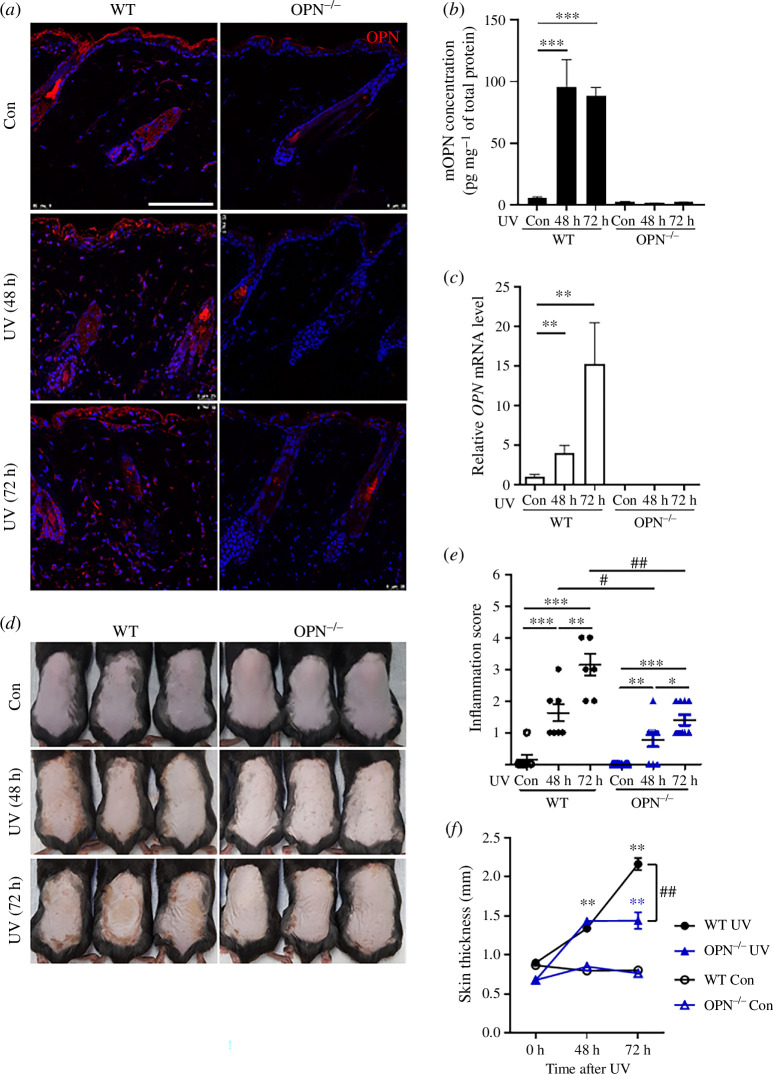
UV-induced skin inflammation is decreased in OPN knockout mice. WT and OPN^−/−^ male mice (8-week-old, *n* = 5) were irradiated with 200 mJ cm^−2^ UVA and UVB and skin tissues were analysed after 48 h and 72 h. (*a*) Immunofluorescence staining of OPN (red) and nuclei (using DAPI; blue) in skin sections. Scale bars, 100 μm. (*b*) OPN levels in the whole skin tissue 48 and 72 h after UV exposure analysed using ELISA. (*c*) *OPN* mRNA expression in whole skin tissue at the indicated timepoints after UV irradiation compared with the control analysed using qRT-PCR. (*d*) Representative images of mice skin with and without UV exposure. (*e*) Inflammation severity score of mouse skin, graded by referring to photographs: 0 = none, 1 = mild, 2 = moderate, 3 = severe and 4 = very severe. (*f*) Skin thickness was measured using calipers at the indicated timepoints. Data are presented as mean ± standard errors of mean, *, ** and *** indicate *p *< 0.05, *p *< 0.01 and *p *< 0.001 versus non-irradiated control in each mouse strain, respectively; # and ## indicate *p *< 0.05 and *p *< 0.01 versus between groups at each time point, respectively.

As in human skin, immunofluorescence staining of skin sections from mice showed that OPN was expressed in the epidermis and dermis of wild-type (WT) mice, albeit at low levels (figure 2*a*). However, the expression of OPN increased in dermal appendages, including in hair follicles, sebaceous glands, blood vessels and the epidermal layer, 48 and 72 h after UV irradiation. Consistent with this finding, increased protein levels (figure 2*b*) and mRNA expression (figure 2*c*) of OPN were observed in the whole skin of WT mice using enzyme-linked immunosorbent assay (ELISA) and quantitative real-time polymerase chain reaction (qRT-PCR), respectively. The expression of OPN increased significantly in both the epidermis and dermis 48 and 72 h after UV irradiation. As expected, OPN was not expressed in OPN^−^**^/−^** mice (figure 2*a–c*).

Following UVA and UVB irradiation (200 mJ cm^–2^), UV-induced erythema and scabs were observed to be less severe in OPN^−^**^/^**^−^ mice (figure 2*d*,*e*). The inflammation severity was scored on a scale of 0 (none) to 4 (very severe) based on visual inspection, and the results showed that WT mice had mean scores of 1.6 ± 0.4 at 48 h and 3.1 ± 0.4 at 72 h, while OPN^−^**^/−^** mice showed a significantly decreased inflammation severity, with mean scores of 0.8 ± 0.3 at 48 h and 1.4 ± 0.3 at 72 h, respectively. Furthermore, skin thickening at 72 h after UV irradiation was reduced by 33.4 ± 5.5% in OPN^−^**^/−^** mice compared with in WT mice (figure 2*f*), which was significant. These skin phenotypes suggested that OPN played a role in augmenting UV-induced responses.

*p*-values were calculated using the Mann–Whitney *U*-test. WT, wild-type; UV, ultraviolet; OPN, osteopontin; DAPI, 4′,6-diamidino-2-phenylindole; ELISA, enzyme-linked immunosorbent assay; qRT-PCR, quantitative real-time polymerase chain reaction.

### 3.3. OPN promotes UV-induced production of inflammatory cytokines and proteolytic enzymes in mouse skin

**Figure 3.**
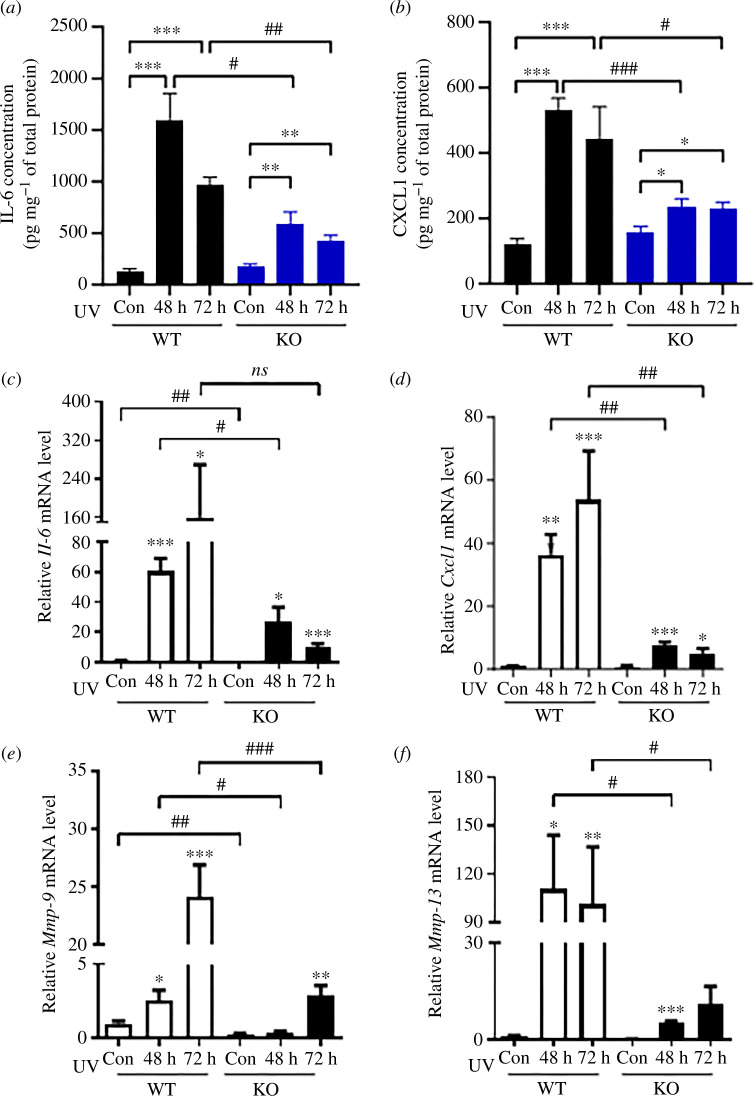
OPN is required for the expression of IL-6, CXCL1, MMPs in UV-irradiated mouse skin. Skin tissues were obtained from WT and OPN^−^**^/−^** mice after 48 and 72 h of UVA and UVB irradiation (200 mJ cm^−^**^2^**). (*a*,*b*) Protein levels of mouse IL-6 (*a*) and CXCL1 (*b*) determined by ELISA. (*c–f*) mRNA expression of *Il-6* (*c*), *Cxcl1* (*d*), *Mmp-9* (*e*) and *Mmp-13* (*f*) in mouse skin determined by qRT-PCR. All data are presented as mean ± standard errors of mean. *, ** and *** indicate *p *< 0.05, *p *< 0.01 and *p *< 0.001 versus non-irradiated control in each mouse strain, respectively; #, ## and ### indicate *p *< 0.05, *p *< 0.01, *p *< 0.001 versus between groups at each time point, respectively. *p*-values were calculated using the Mann–Whitney *U*-test. WT, wild-type; UV, ultraviolet; OPN, osteopontin; ELISA, enzyme-linked immunosorbent assay; qRT-PCR, quantitative real-time polymerase chain reaction; IL, interleukin; CXCL-1, chemokine (C-X-C motif) ligand 1; MMP, matrix metalloproteinase.

Next, the ability of WT and OPN^−^**^/−^** mice to produce inflammatory cytokines in response to UVA and UVB irradiation (200 mJ cm^−2^) were compared. OPN promotes the expression of interleukin (IL)-6 and chemokine (C-X-C motif) ligand 1 (CXCL1), which play vital roles in skin inflammation [21]. An ELISA assay revealed that the protein levels of IL-6 and CXCL1, which increased significantly in the skin upon UV irradiation, were significantly reduced in OPN^−^**^/−^** mice compared with in WT mice (figure 3*a*,*b*). Consistent with this finding, in the UV-exposed skin of WT mice, there was a significant increase in the mRNA levels of *Il-6* and *Cxcl1*; by contrast, the expression levels of both cytokines in the skin of OPN^−^**^/−^** mice were significantly lower than that in the skin of WT mice 48 h after UV irradiation (figure 3*c*,*d*).

In addition to inflammatory cytokines, UV light induces the expression of matrix metalloproteinases (MMPs), including MMP-9 (gelatinase) and MMP-13 (collagenase in mice), in the skin, which mediate ECM degradation in the dermis. Therefore, the mRNA levels of *Mmp-9* and *Mmp-13* were analysed, and it was found that the mRNA levels of *Mmp-9* and *Mmp-13* were significantly lower in the skin of OPN^−^**^/−^** mice than in that of WT mice following UV irradiation (figure 3*e*,*f*). These results indicate that OPN may increase the inflammation induced by UV irradiation.

### 3.4. OPN mediates UV-induced infiltration of inflammatory cells into the skin

**Figure 4.**
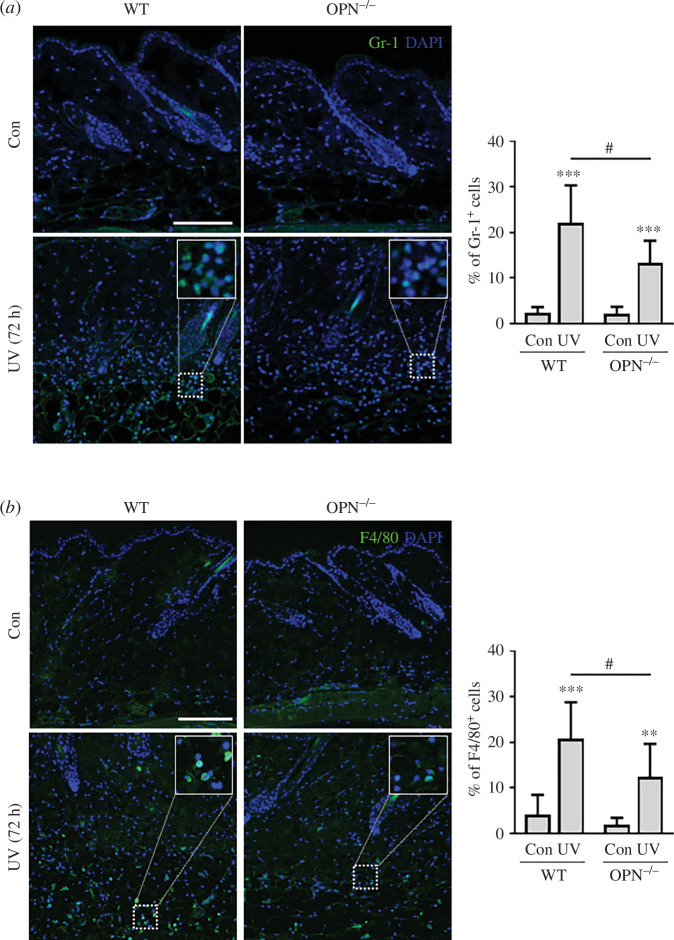
OPN^−/−^ mice show a decrease in the number of inflammatory cells in the skin in response to UV irradiation. WT and OPN^−/−^ male mice (8-week-old, *n* = 5) were irradiated with 200 mJ cm^−2^ UVA and UVB and skin tissue were analysed after 72 h. (*a*) Skin sections stained with Gr-1 antibody (green) and DAPI (blue). Gr-1-positive cells were counted and shown as percentages relative to DAPI-positive cells. (*b*) Representative immunofluorescent images of F4/80 staining. F4/80-positive cells (green) are presented as percentages relative to DAPI-positive cells (blue). All results are represented as a mean ± SEM. ** and *** indicate *p *< 0.01 and *p *< 0.001 versus corresponding control, respectively; # indicates *p *< 0.05 versus UV-irradiated group. *p*-values were calculated by Mann–Whitney *U*-test. Scale bars, 100 μm. WT, wild-type; UV, ultraviolet; OPN, osteopontin; DAPI, 4′,6-diamidino-2-phenylindole.

Inflammatory cells such as granulocytes and macrophages increase in the skin upon UVA and UVB irradiation, and OPN attracts these cells [22]. Thus, immunofluorescence staining was performed using Gr-1, a granulocyte marker, or F4/80, a macrophage marker. We found that after UV exposure, there was marked decrease of Gr-1-positive cells in the dermis of the OPN^−/−^ mice skin compared with in WT mice skin (figure 4*a*). Moreover, immunofluorescence staining showed that OPN^−^**^/−^** mice had reduced infiltration of F4/80 positive cells into the dermis of the UV-irradiated skin (figure 4*b*) compared with WT mice. These results indicate that OPN may be a major factor that mediates inflammation by attracting inflammatory cells.

### 3.5. hOPN has pro-inflammatory properties in the mouse skin

**Figure 5.**
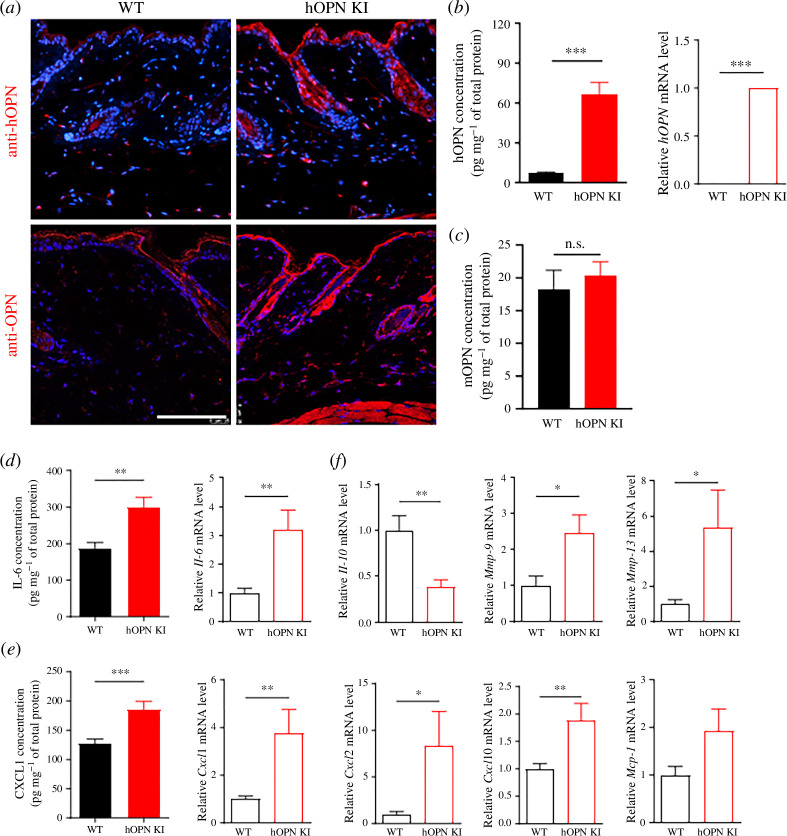
hOPN KI mice show increased inflammation in the skin. Skin tissues were obtained from 8-week-old WT and hOPN KI mice (female WT, *n* = 10; KI, *n* = 8; male WT, *n* = 8; KI, *n* = 5). (*a*) Immunofluorescence staining with anti-OPN or anti-hOPN (red) and DAPI (blue) in skin sections of male mice. Scale bars, 100 μm. (*b*) The hOPN concentration in skin tissue analysed by ELISA, and its mRNA levels analysed by qRT-PCR. (*c*) mOPN protein expression in skin tissues analysed using ELISA. (*d*,*e*) Protein and mRNA levels of mouse IL-6 (*d*) and CXCL1 (*e*). (*f*) mRNA expression levels of *Il-10, Mmp-9, Mmp-13, Cxcl2, Cxcl10* and *Mcp-1* in mouse skin determined by qRT-PCR. Data are presented as mean ± standard errors of mean, *, ** and *** indicate *p *< 0.05, *p *< 0.01 and *p *< 0.001, respectively. *P*-values were calculated using the Mann–Whitney *U*-test. WT, wild-type; UV, ultraviolet; OPN, osteopontin; DAPI, 4′,6-diamidino2-phenylindole; hOPN, human OPN; mOPN, mouse OPN; KI, knockin. IL, interleukin; CXCL, chemokine (C-X-C motif) ligand; MMP, matrix metalloproteinase; ELISA, enzyme-linked immunosorbent assay; qRT-PCR, quantitative real-time polymerase chain reaction.

To investigate whether increased OPN levels induced skin inflammation, the skin of human OPN knockin (hOPN KI) mice without UV stimulation was used. First, mice OPN (mOPN) and hOPN expression were investigated in the skin of hOPN KI and WT mice. Staining of skin tissue with anti-hOPN antibodies confirmed that hOPN was expressed only in hOPN KI mice (figure 5*a*). Similarly, ELISA and qRT-PCR assays showed that hOPN was highly expressed only in the hOPN KI mice (figure 5*b*). In addition, staining with an anti-OPN antibody that bind to both hOPN and mOPN showed a noticeable increase in the signals in the skin of hOPN KI mice (figure 5*a*). There was no change in mOPN protein expression between WT and hOPN KI mice (figure 5*c*).

Changes in the expression of inflammatory cytokines were investigated to determine whether inflammation was increased in the skin of hOPN KI mice. In the skin of hOPN KI mice, the concentrations of IL-6 and CXCL1, as measured by ELISA, were significantly higher than that in the skin of WT mice, and the expression of each gene was significantly increased (figure 5*d*,*e*). In addition, the expressions *of Il-10, Mmp-9, Mmp-13, Cxcl2, Cxcl10* and *Mcp-1* mRNAs, which are inflammatory modulators or mediators in the skin, were compared between the skins of hOPN KI and WT mice. The expression of *Il-10*, an anti-inflammatory cytokine, was noticeably reduced, whereas other pro-inflammatory factors were highly expressed in the skin of hOPN KI mice (figure 5*f*). These results suggest that OPN is directly related to the increased expression of inflammatory cytokines in the skin.

### 3.6. Treatment of recombinant OPN induces MMP-1 production in normal human dermal fibroblasts (NHDF) and normal human epidermal keratinocytes (NHEK)

**Figure 6.**
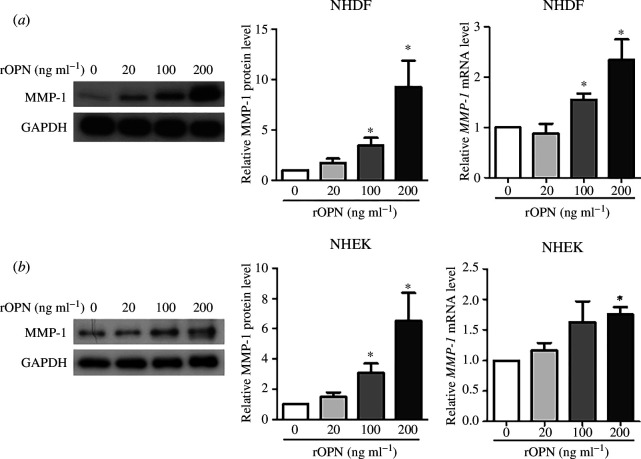
rOPN significantly increases MMP-1 expression. Primary NHDFs and NHEKs were treated with rOPN (20, 100 and 200 ng ml^−1^) or vehicle (0 ng ml^−1^, PBS with 0.1% BSA) for 72 h. The cells were harvested after 24 h for mRNA analysis and after 72 h for protein analysis. The protein levels of MMP-1 in (*a*) NHDFs and (*b*) NHEKs were analysed by western blotting, and the band intensity was evaluated using ImageJ software. The protein levels of MMP-1 in conditioned media were normalized to those of GAPDH in the cell lysates. Relative protein levels were compared with those of vehicle-treated control samples (0 ng ml^−1^). Data are presented as mean ± standard errors of mean (*n* = 3). * indicates *p *< 0.05. *p*-values were calculated using Student’s *t*‐test. NHDF, normal human dermal fibroblasts; NHEK, and normal human epidermal keratinocytes; rOPN, recombinant osteopontin; PBS, phosphate-buffered saline; BSA, bovine serum albumin; MMP, matrix metalloproteinase; GAPDH, glyceraldehyde 3-phosphate dehydrogenase.

When human skin is exposed to UV light, dermal fibroblasts and epidermal keratinocytes secrete MMP-1, an interstitial collagenase that breaks down collagen and contributes to photoaging. To investigate the effect of OPN on the expression of MMP-1, primary NHDFs and NHEKs were treated with recombinant human OPN (rOPN). rOPN was added to the cell culture medium at concentrations of 0 (control), 20, 100 and 200 ng ml^−1^ and MMP-1 protein expression in the medium was evaluated by western blotting after 72 h. rOPN increased the expression of MMP-1 in a dose-dependent manner in both NHDFs and NHEKs. MMP-1 protein levels were 1.7 ± 0.4, 3.4 ± 0.8 and 9.3 ± 2.6 fold higher compared with the control in NHDFs (figure 6*a*) following treatment with indicated concentrations of rOPN, respectively. Similarly, in NHEKs, following treatment with indicated concentrations of rOPN, MMP-1 levels were 1.5 ± 0.3, 3.1 ± 0.5 and 6.5 ± 1.6 fold higher compared with the control, respectively (figure 6*b*).

In addition, the mRNA expression of MMP-1 was investigated in cells 24 h after treatment with rOPN. MMP-1 mRNA levels significantly increased at rOPN concentrations of 100 and 200 ng m−1 in fibroblasts (figure 6*a*) and at a rOPN concentration of 200 ng ml in keratinocytes (figure 6*b*). These findings suggest that an increase in OPN levels in the skin may contribute to the induction of MMP-1 expression.

### 3.7. Treatment with rOPN increases inflammatory cytokine expression in skin cells

**Figure 7.**
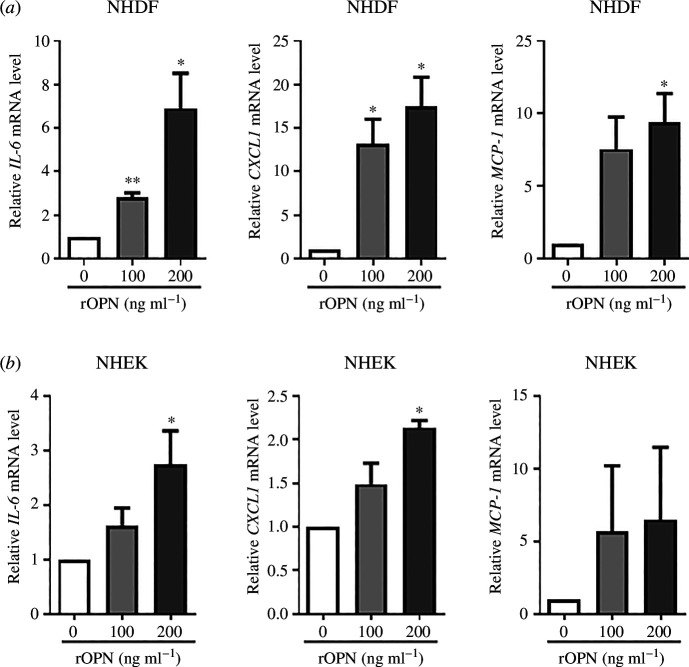
rOPN treatment induces inflammatory cytokines in NHDF and NHEK. NHDFs and NHEKs were treated with rOPN (100 or 200 ng ml^−1^) or vehicle (PBS with 0.1% BSA) for 24 h. The total RNA was extracted, and the mRNA levels of *Il-6*, *Cxcl1* and *Mcp-1* were measured by qRT-PCR and normalized to the expression level of *36B4* in (*a*) NHDFs (*n* = 4) and (*b*) NHEKs (*n* = 4). Data are presented as mean ± standard errors of mean from four independent experiments, * and ** indicate *p *< 0.05 and *p *< 0.01 versus controls, respectively. Statistical comparisons were performed using the Student’s *t*‐test. NHDF, normal human dermal fibroblasts; NHEK, and normal human epidermal keratinocytes; rOPN, recombinant osteopontin; PBS, phosphate-buffered saline; BSA, bovine serum albumin; IL, interleukin; CXCL, chemokine (C-X-C motif) ligand; qRT-PCR, quantitative real-time polymerase chain reaction.

In mice experiments, the expression of inflammatory cytokines was influenced by OPN. Thus, the effect of rOPN treatment on the expression of inflammatory cytokines was assessed *in vitro*. Primary NHDFs and NHEKs were treated with 0 (control), 20, 100 and 200 ng ml^−1^ of rOPN for 24 h and the cells were harvested. As shown in figure 7*a*, rOPN significantly induced *Il-6*, *Cxcl1* and *Mcp-1* mRNA expression in NHDFs in a dose-dependent manner up to concentrations of 200 ng ml^−1^. In addition, the treatment of NHEKs with 200 ng ml^−1^ rOPN significantly increased *Il-6* and *Cxcl1* mRNA expression at 24 h (figure 7*b*). These results suggest that OPN plays a role in increasing the levels of inflammatory cytokines.

## 4. Discussion

OPN is a multifunctional protein with cytokine-like properties that plays pro- and anti-inflammatory roles in various physiological and pathological processes, including tumorigenesis and inflammation [23]. Induction of skin inflammation by UV irradiation is well documented and is known to cause skin disorders such as photoaging and cancer by altering tissue homeostasis [24,25]. However, the exact role of OPN in the skin has not yet been elucidated. Herein, we investigated the effects of OPN expression on UV-induced skin responses and inflammation.

Herein, we examined OPN expression in the skin after UV irradiation. In human skin tissues irradiated with UV (2 MED), OPN protein expression markedly increased after 24, 48 and 72 h. This is consistent with a previous study showing that OPN expression in the human skin is elevated by sunlight and is involved in skin cancer [26]. UV radiation is detrimental to the skin and causes inflammation, and OPN is known to be involved in inflammation [23,25].

Therefore, to investigate whether OPN is directly involved in UV-induced skin inflammation, we compared responses of the skin to UV irradiation in WT mice and genetically modified mice lacking OPN. It was observed that OPN expression significantly increased only in the skin of WT mice 48 and 72 h after acute UV irradiation. In addition, UV irradiation increased inflammatory symptoms such as skin thickening, erythema, oozing, scab and wound, which were generally lower in OPN^−/−^ mice than in WT mice.

Moreover, the secretion of inflammatory cytokines, such as IL-6 and CXCL1, was alleviated in the skin of mice lacking OPN. Furthermore, the number of granulocytes and macrophages infiltrating the dermis of the UV-irradiated skin was lower in OPN^−/−^ mice than in WT mice. This is thought to be because cytokines such as IL-6 and CXCL1 are associated with macrophage polarization [27] and attraction of neutrophils to inflammatory sites [28,29]. These cytokines were selected due to their relevance in UV-induced skin inflammation and their established roles in OPN-mediated immune responses [30]. Existing literature suggests that keratinocytes, fibroblasts and infiltrating immune cells are the main sources of IL-6 and CXCL1 [31,32]. Sica *et al*. reported that macrophages in the wound site of OPN^−/−^ mice exhibited high expression of mannose receptors, which are associated with reduced expression of inflammatory cytokines such as IL-6 and tumour necrosis factor-α [33]. IL-6 has been shown to be a key molecule in acute and chronic inflammation and its level is elevated in inflammatory diseases [34]. CXCL1 also plays an important role in inflammatory responses in the skin and contributes to the development of skin tumours [35]. OPN also acts as a chemoattractant for various immune cells, including neutrophils, macrophages, monocytes and T cells [30,36]; the autocrine secretion of sOPN by macrophages may also increase their ability to invade inflamed tissues [37]. Therefore, it is considered that OPN elevation due to UV irradiation further increases the number of inflammatory cells in the irradiated skin. This may have contributed to the increased numbers of infiltrating macrophages and granulocytes observed in our study. Based on these results, it could be suggested that the recruitment of inflammatory cells by OPN might play a role in allowing more inflammatory cells to invade the skin tissue, thereby further increasing the amounts of inflammatory cytokines. Further research using IF staining is necessary to localize the exact sources of these cytokines in the context of UV-induced inflammation and OPN modulation to provide a more detailed understanding of the cellular mechanisms involved.

Interestingly, in the skin of OPN^−/−^ mice, the UV-induced expression of MMP-9 and MMP-13 was significantly suppressed compared with that in the skin of WT mice. MMPs are modulated by pro-inflammatory cytokines and are highly expressed during wound healing, angiogenesis, inflammation, skin ageing and cancer [38]. MMPs are enzymes that degrade the extracellular matrix in the skin; MMP-1 (collagenase) decomposes type I and type III collagen, MMP-9 (gelatinase B) decomposes gelatin, type IV and type V collagen, and MMP-13 (collagenase) decomposes type II collagen [39,40]. In a study regarding whether OPN regulated MMP expression, OPN was observed to promote the migration of human chondrosarcoma cells by upregulating MMP-9 via nuclear factor-κB-dependent pathways [41]. In addition, Xu *et al*. reported that the treatment of chondrocytes with OPN increased the mRNA and protein levels of MMP-13, which breaks down matrix components and increases cartilage degeneration, contributing to the development of osteoarthritis [42]. Therefore, OPN contributes to UV-induced skin responses and inflammation by directly or indirectly increasing MMPs expression.

By contrast, to confirm whether OPN overexpression had the opposite effect of OPN deficiency, experiments with hOPN KI mice were conducted. These were transgenic mice in which the hOPN gene was inserted into the ROSA26 locus to induce high expression throughout the body. Only 63% of the amino acid sequences of human and murine OPNs are identical [43]. However, OPN has a functional Arg-Gly-Asp (RGD) domain that is highly conserved between humans and rodents, which binds to integrins such as αvβ1, αvβ3, αvβ5, αvβ6, α5β1 and α8β1 integrins [44]. C57BL/6 mice with the human OPN gene inserted had no differences in size, reproductive ability or abnormalities compared with WT mice; however, they had obesity-induced clinical problems similar to a diet-induced obesity mouse model [43].

A comparison of the expression of pro-inflammatory cytokines and MMPs between hOPN KI and WT mice showed that the expression levels of these factors were higher in hOPN KI mice than in WT mice without UV irradiation. When human NHDFs and NHEKs were treated with OPN, the protein and mRNA expression of MMP-1 increased in a dose-dependent manner, and the mRNA expression of inflammatory cytokines such as *Il-6, Cxcl1* and *Mcp-1* also increased 24 h after treatment. These results suggest that OPN is directly related to the increased expression of inflammatory factors in the skin.

However, the signalling processes involved in the inflammatory cytokine response or induction of MMPs were not elucidated in this study. OPN is known to activate the p38 mitogen-activated protein kinase signalling pathway [45,46], which is involved in the regulation of MMP expression and UV-induced skin responses. Therefore, it is necessary to investigate the exact function of OPN through mechanistic studies. Additionally, future studies may explore additional cytokines influenced by OPN to further understand its role in skin inflammation and repair mechanisms. In addition to acute UV irradiation, it would also be useful to investigate the long-term effects of OPN expression on the skin by comparing skins of aged OPN^−/−^ mice with that of WT mice.

Collectively, these results suggest that OPN is a potential target for the attenuation of skin inflammation and skin phenotypes in response to UV irradiation.

## Data Availability

This article has no additional data.
